# Supplementary material for: The structural basis of promiscuity in small multidrug resistance transporters
Source: Nat Commun. 2020 Nov 27;11:6064. doi: 10.1038/s41467-020-19820-8 (PMC7695847; doi:10.1038/s41467-020-19820-8)
Supplement: Supplementary file 1 — Supplementary Information [file 41467_2020_19820_MOESM1_ESM.pdf]

Supplementary Information:

The structural basis of promiscuity in Small Multidrug Resistance transporters

Ali A. Kermani, Christian B. Macdonald, Olive E. Burata, B. Ben Koff, Akiko Koide, Eric Denbaum, Shohei Koide, Randy B. Stockbridge

Supplementary Figure 1. SMR sequence similarity networks with additional annotation. A. Full sequence similarity network with gene annotation of each cluster, where known. B. Distribution of SMR proteins in archaea and different bacterial taxa. Sequence similarity network as in Figure 1A with coloring according to taxa as indicated.

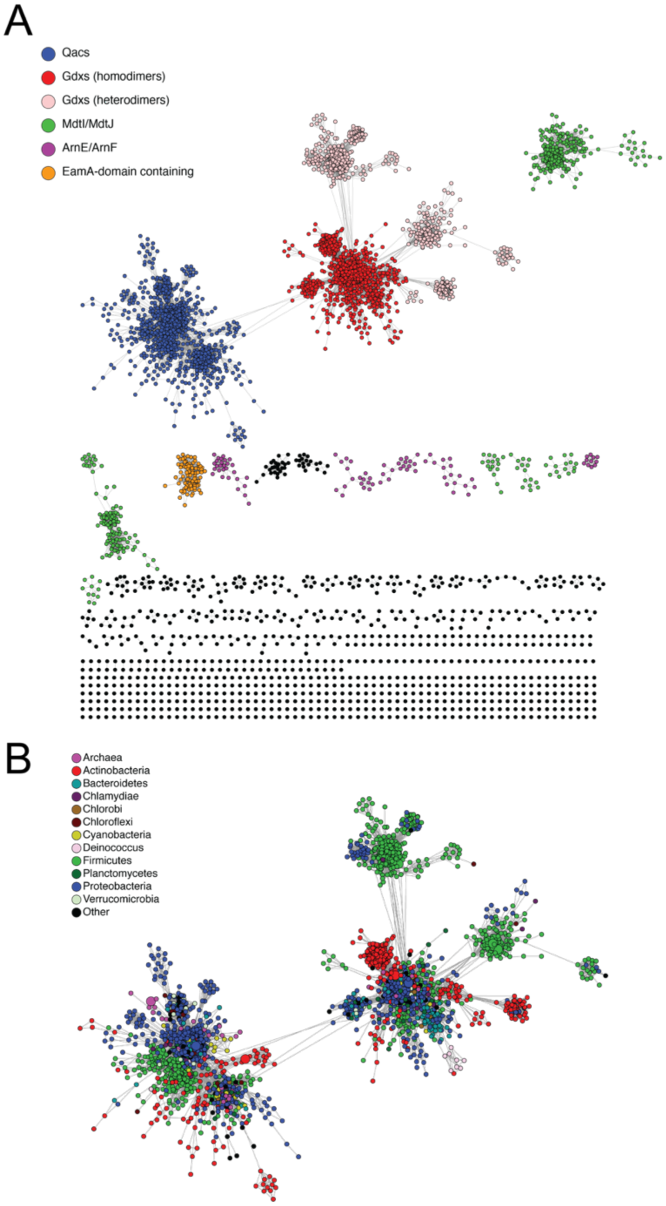

Supplementary Figure 2. Sequence alignment of functionally characterized homodimeric SMRs [1-14].

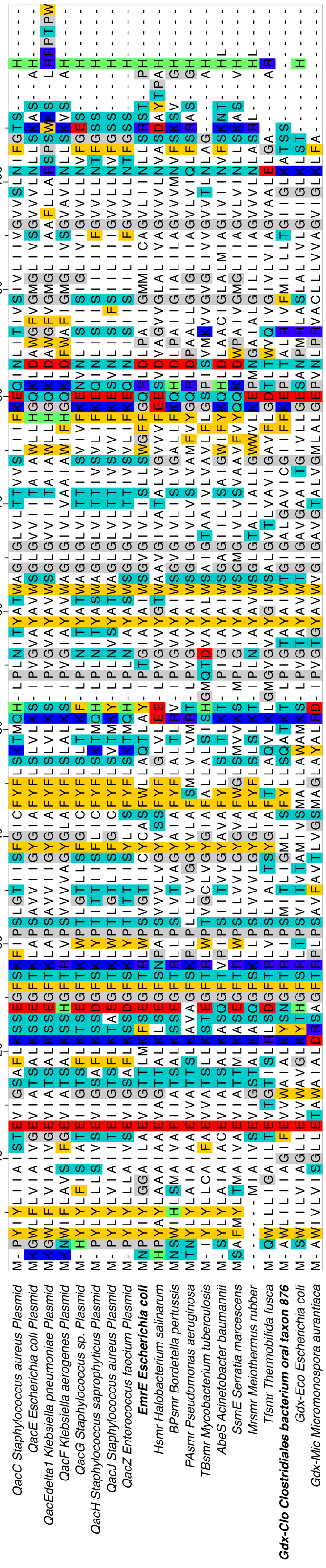

Supplementary Figure 3. Representative SSM electrophysiology recordings. Currents elicited after perfusion with substrate are shown for empty liposomes (left; black), Gdx-Clo proteoliposomes (middle; blue) and EmrE proteoliposomes (right; red).

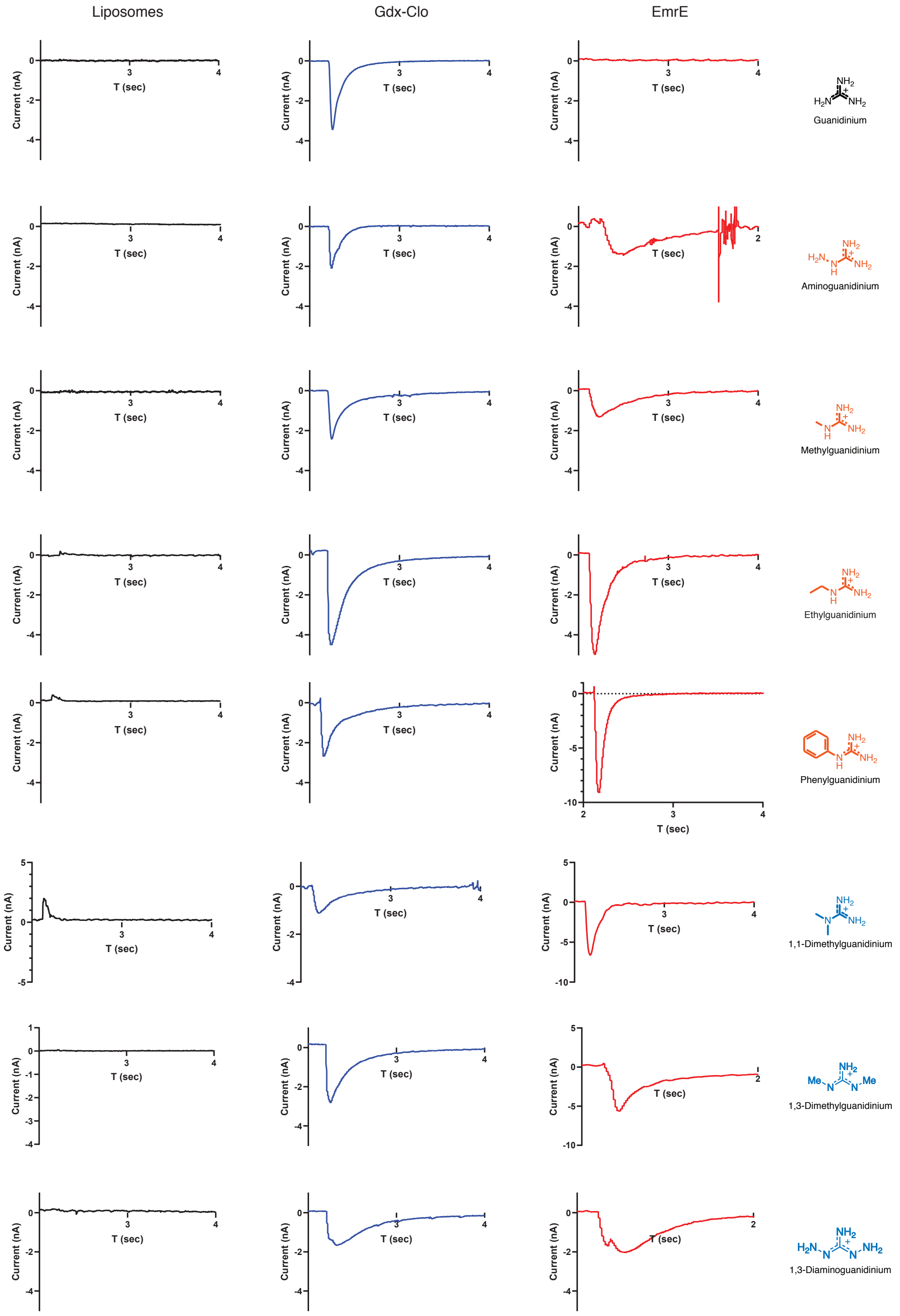

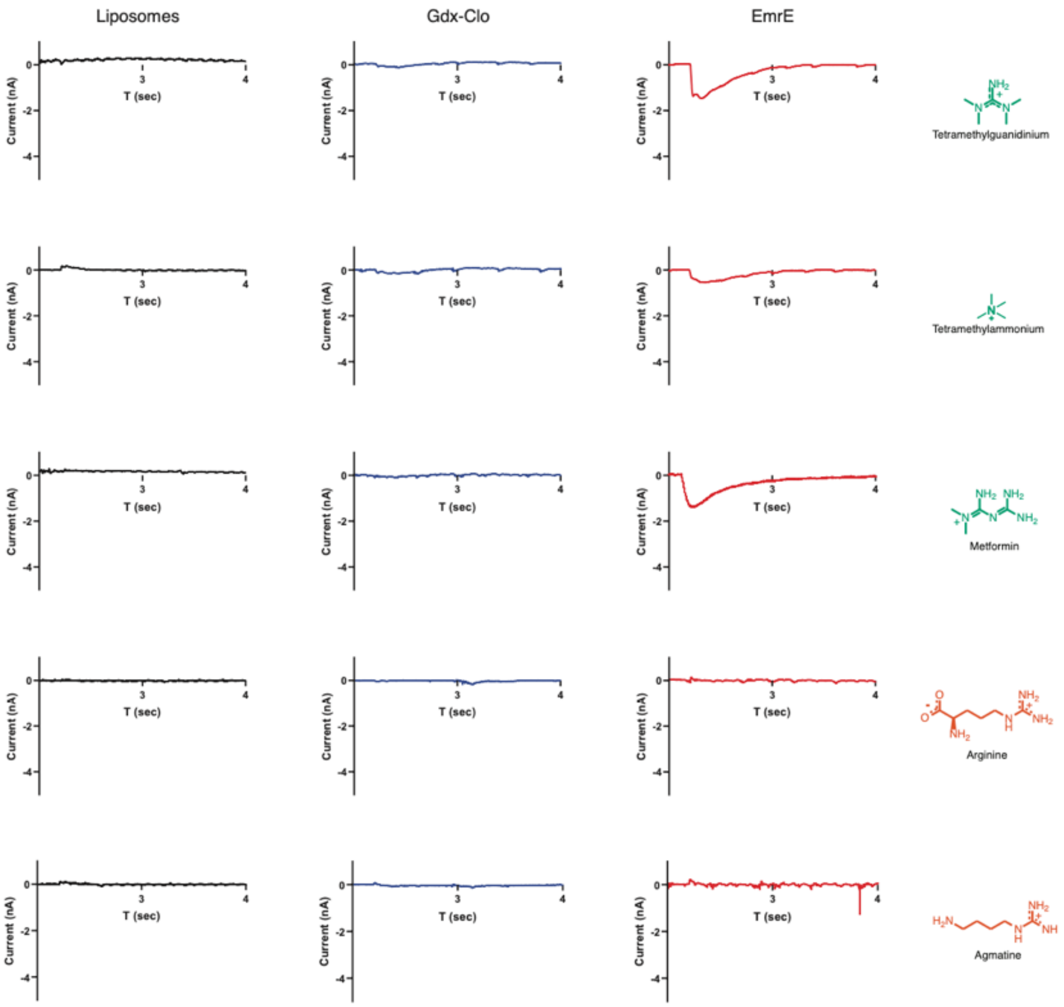
Liposomes
Gdx-Clo
EmrE
Current (nA)
T (sec)
Tetramethylguanidinium
Tetramethylammonium
Metformin
Arginine
Agmatine

Supplementary Figure 4. SSM traces for tetraphylphosphonium (TPP^+, top) and ethidium ($EtBr^+$) perfusion of liposomes that do not contain protein.

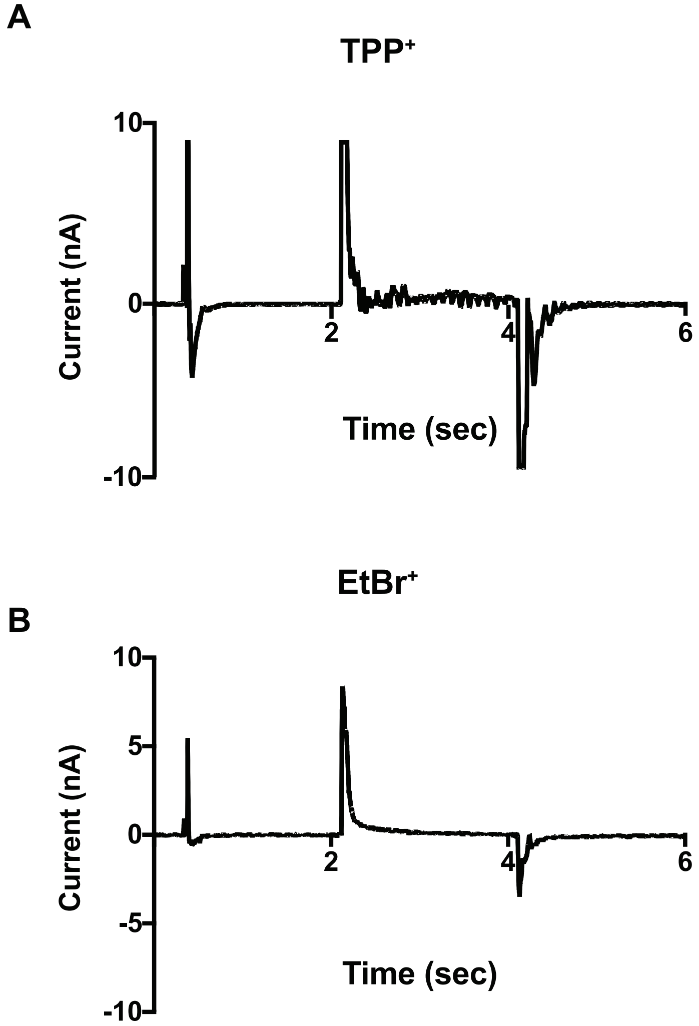

Supplementary Figure 5. Uptake of ^{14}C Gdm^{+} into Gdx-Clo proteoliposomes in exchange for the indicated substrate. Experiment performed as in main text Figure 2, with fractional uptake measured relative to total radioactive counts in reaction mixture. Error bars represent the mean and SEM of three technical replicates.

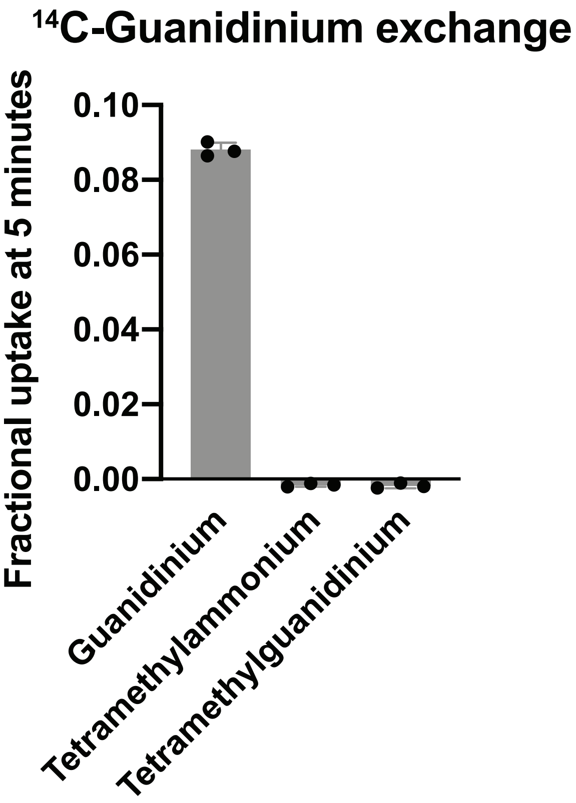

Supplementary Figure 6. Venn diagram showing overlapping transport specificities of Gdx-Clo and EmrE.

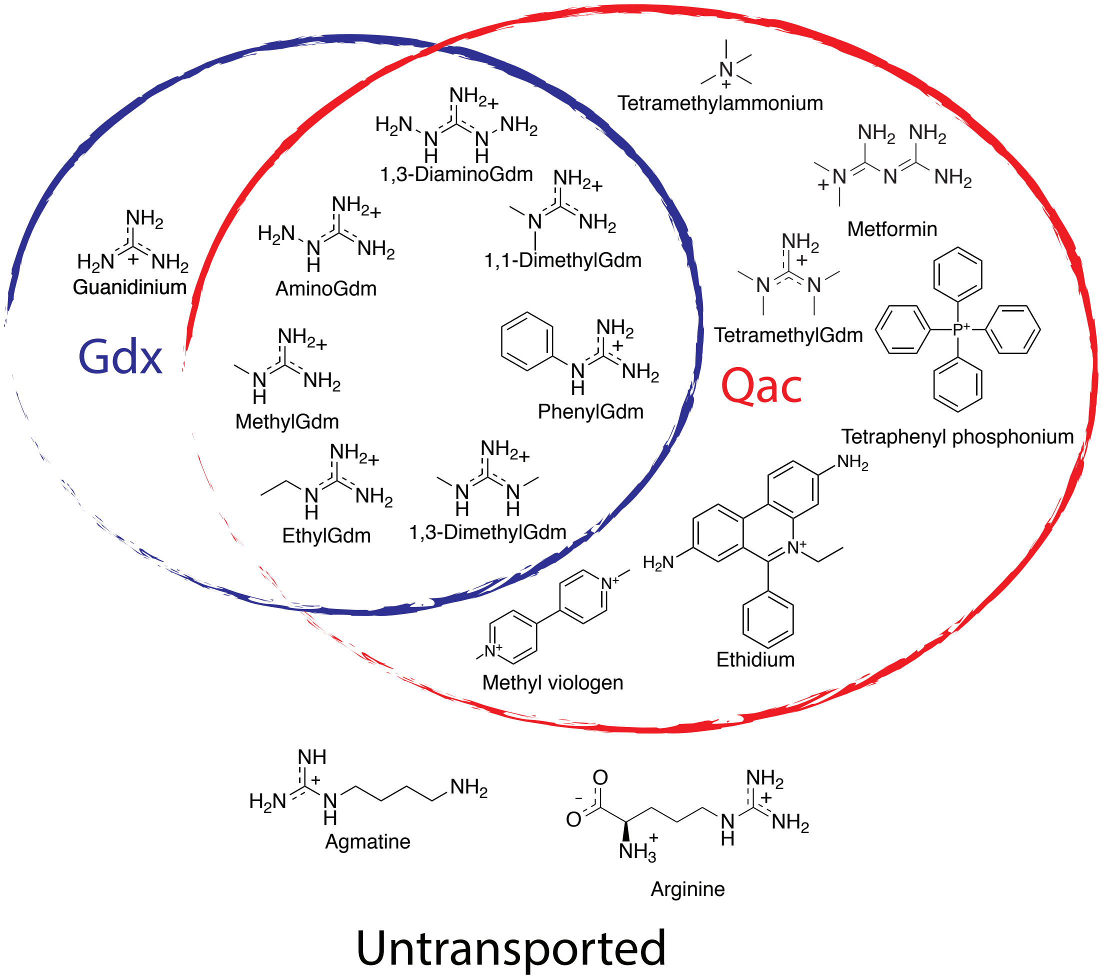

Supplementary Figure 7. Experimental electron density maps for Gdx-Clo. A. Cartoon view of one subunit from Gdx-Clo, with the solvent-flattened electron density map calculated from SHARP contoured at 1.3σ (teal), and anomalous difference density from seleno-L-methionine contoured at 5σ (magenta). B. Electron density map for one subunit of Gdx-Clo (octylGdm$^+$-bound structure) contoured at 1.8σ.

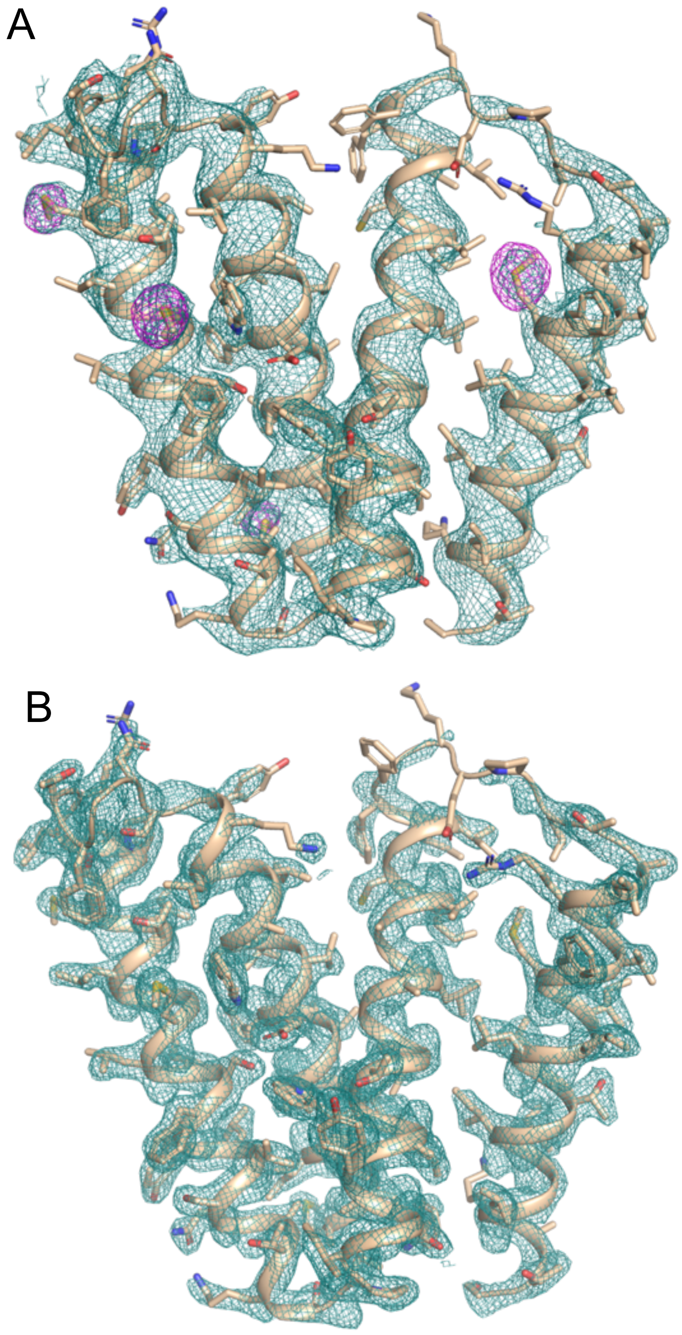

Supplementary Figure 8. Binding interface between monobody Clo-L10 and Gdx-Clo. Gdx-Clo shown in tan and cyan; monobody in green. Residues within H-bonding distance are shown as sticks, with H-bond interactions shown as dashed lines.

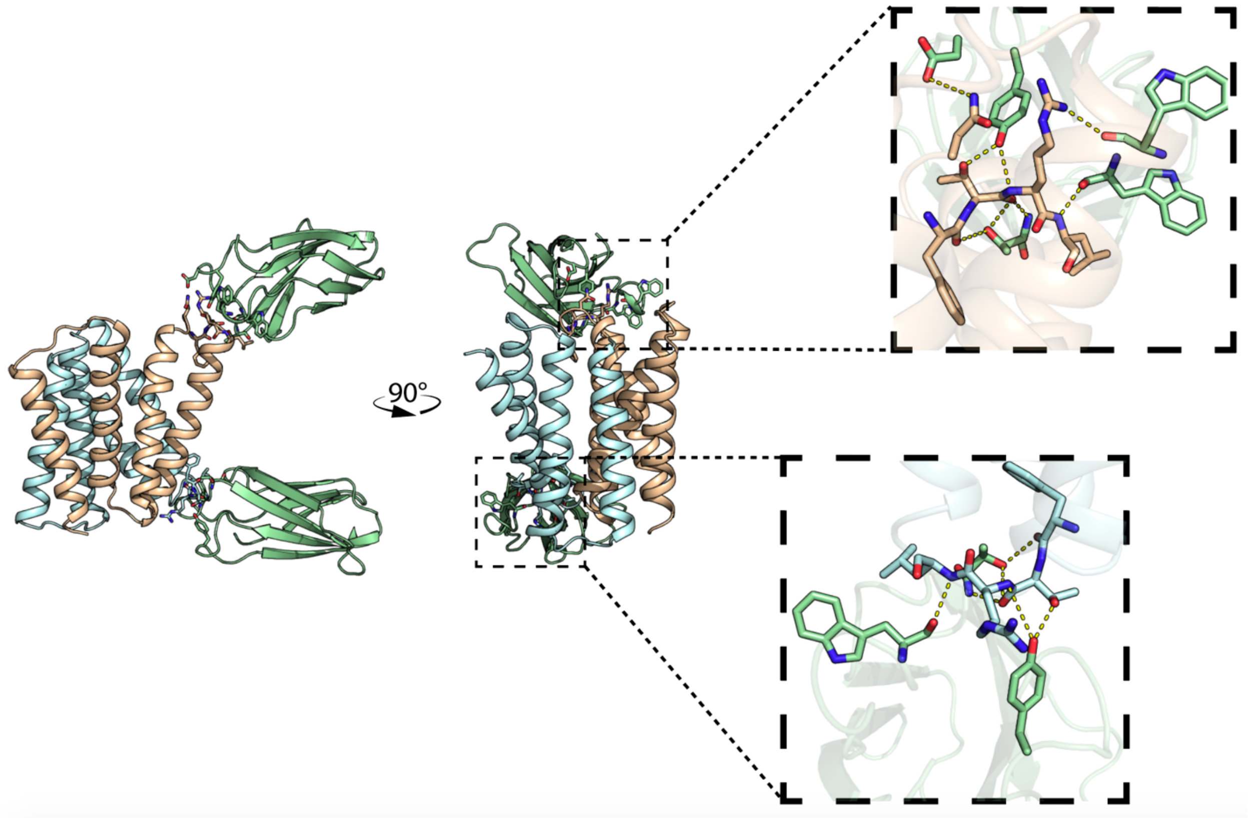

Supplementary Figure 9. Currents mediated by Gdx-Clo in the presence and absence of monobody L10. A. Currents elicited by perfusion with 1 mM Gdm^+. For these experiments, benchmark current values for Gdx-Clo proteoliposomes were measured first (dark blue trace). Second, Gdm^+ was removed by perfusion and L10 monobody was added and incubated with proteoliposomes for three minutes. Third, proteoliposomes were perfused with 1 mM Gdm^+ containing L10 monobody (red trace). Fourth, both Gdm^+ and L10 monobody were perfused away and proteoliposomes were incubated in buffer without L10 monobody for three minutes. Finally, a third recording was collected upon perfusion with 1 mM Gdm^+ (light blue trace) to ensure that currents returned to the benchmark value. B. Fractional inhibition of Gdm^+ currents by L10 monobody, added to the indicated concentrations. Error bars represent the mean and SEM. Data collected from at least three independent sensor preparations derived from two independent protein preparations.

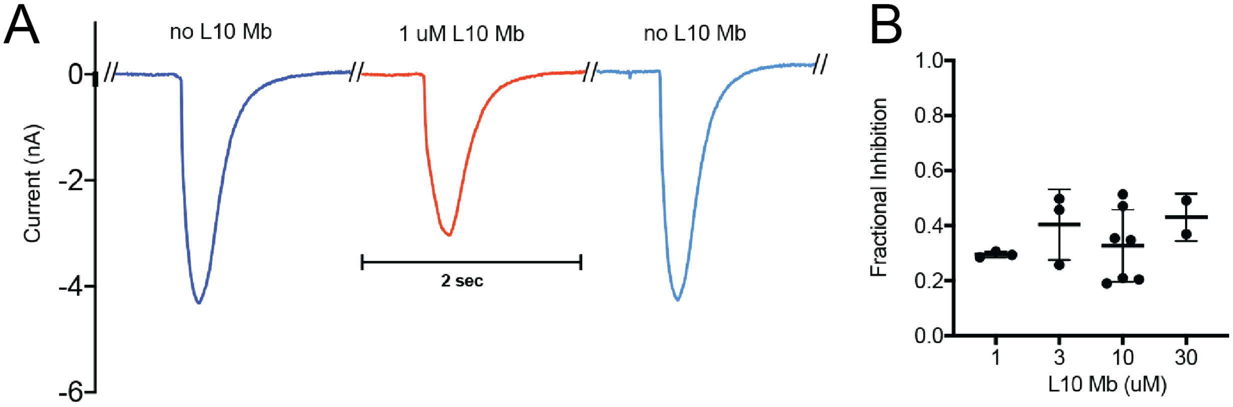

Supplementary Figure 10. Electron density between E13 and E13' in 3.2 Å structure solved with 10 mM Gdm$^+$. Top-down view of Clo-Gdx with subunits colored tan and light blue and E13 sidechains shown as sticks. Fo-Fc map in the region of the E13 sidechains is contoured at 3.2 σ.

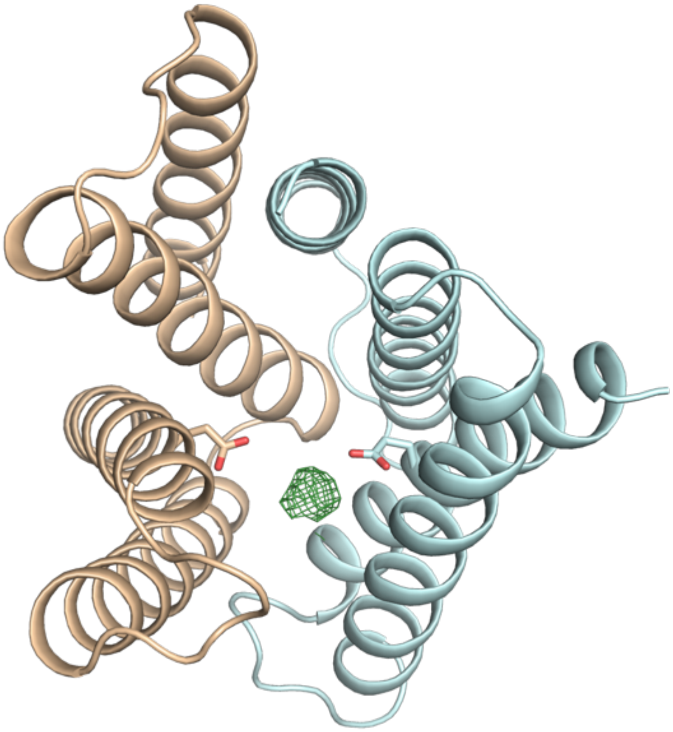

Supplementary Figure 11. Surface rendering of exposed TM3 GxxxG motifs. Left, surface view of Gdx-Clo viewed through the plane of the membrane. Right, view is rotated 90° and tilted to view the interior of the aqueous-exposed vestibule. Coloring of surface rendering corresponds to TM3 sequence shown below. The first (magenta) GxxxG motif is exposed to the membrane in subunit A, and packed in the protein interior in subunit B. The second (dark blue) GxxxG motif is exposed to the aqueous vestibule in subunit B, but packed in the protein interior in subunit A. Conformational exchange swaps the accessibility of each GxxxG motif.

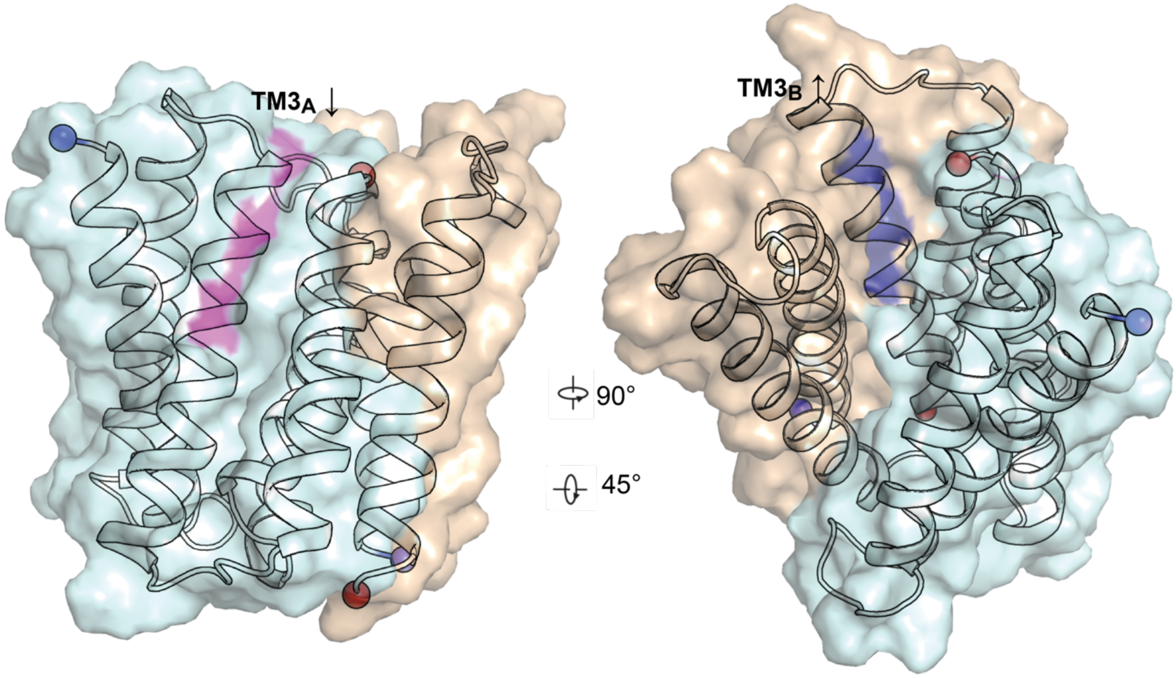

I_{55}GTAYAIWTGI_{65}GALGAVICG

Supplementary Figure 12. Structural alignment of Gdx-Clo (subunits in tan and light blue as in main text) and CMP-sialic acid transporter from the SLC35 family (PDB: 6I1R [https://www.rcsb.org/structure/6i1r]; shown in dark gray with helix insertions in green). SLC35 proteins that share this fold have been structurally characterized, including [15-17]. Left panel: top-down view of structural alignment. The helices are numbered for Gdx-Clo. Right panels: surface representation viewed through plane of membrane, with approximate membrane boundaries shown.

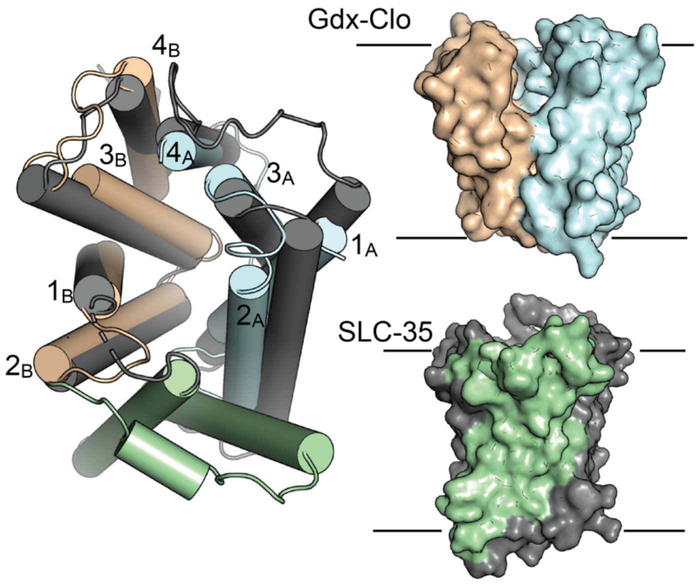

Table S1. Data collection, phasing and refinement statistics for Gdx-Clo complexes

	Clo-L10-Se	Clo-L10-PheGdm$^+$	Clo-L10-OctylGdm$^+$
**Data collection**			
Space group	C121	C121	P1
Cell dimensions			
a, *b*, *c* (Å)	141.8, 51.09, 108.43	141.82, 50.51, 108.58	51.04, 75.83, 109.12
α, β, γ (°)	90, 93.08, 90	90, 92.18, 90	92.54, 90.04, 109.63
Resolution (Å)	54.14-3.2(3.43-3.2)	58.32-2.53 (2.98-2.53)	109.0-2.32 (2.72-2.32)
Ellipsoidal Resolution Limit (best/worst)[a]	N/A	2.53/4.52	2.32/3.89
% Spherical Data Completeness[a]	99.9 (100)	36.5 (4.7)	37.8 (5.0)
% Ellipsoidal Data Completeness[a]	N/A	85.5 (66.2)	85.3 (54.2)
R_{merge}[a]	0.06 (0.15)	0.132 (0.65)	0.074 (0.38)
R_{meas}[a]	--	0.145 (0.72)	0.086 (0.44)
Mn I / σI[a]	17.3 (8.6)	6.4 (2.8)	9.2 (3.0)
Multiplicity[a]	7.5 (7.6)	7.1 (4.9)	3.8 (3.8)
R_{cullis}	91.2%	N/A	N/A
Phasing Power[b]	.592	N/A	N/A
**Refinement**			
Resolution (Å)	44.3-3.5	58.3-2.5	33.0-2.3
No. reflections	10,076	9,018	24,995
R_{work} / R_{free}	25.2 / 27.8	25.7/30.9	24.6/28.6
Ramachandran Favored	93.6	83.6	94.1
Ramachandran Outliers	1.6	2.7	1.7
Clashscore	7.0	13.2	6.9
R.m.s. deviations			
Bond lengths (Å)	0.003	.007	.002
Bond angles (°)	.707	1.53	.585
Coordinates in Protein Databank	6WK5	6WK8	6WK9

[a] Where applicable, values reported are for anisotropically truncated data performed using the Staraniso webserver (Global Phasing). See *Methods* for details.
[b] Phasing Power = rms ($|F_H|$ / ((F_H + F_P)-(F_{PH})))

Supplementary References:

1 Ninio, S. & Schuldiner, S. Characterization of an Archaeal Multidrug Transporter with a Unique Amino Acid Composition. *Journal of Biological Chemistry* **278**, 12000-12005, doi:10.1074/jbc.M213119200 (2003).

2 Ninio, S., Rotem, D. & Schuldiner, S. Functional Analysis of Novel Multidrug Transporters from Human Pathogens. *Journal of Biological Chemistry* **276**, 48250-48256, doi:10.1074/jbc.M108231200 (2001).

3 Rossi, E. D. *et al.* mmr, a Mycobacterium tuberculosis Gene Conferring Resistance to Small Cationic Dyes and Inhibitors. *Journal of Bacteriology* **180**, 6068-6071, doi:10.1128/JB.180.22.6068-6071.1998 (1998).

4 Minato, Y., Shahcheraghi, F., Ogawa, W., Kuroda, T. & Tsuchiya, T. Functional Gene Cloning and Characterization of the SsmE Multidrug Efflux Pump from Serratia marcescens. *Biol. Pharm. Bull.* **31**, 516-519, doi:10.1248/bpb.31.516 (2008).

5 Brill, S., Falk, O. S. & Schuldiner, S. Transforming a drug/H+ antiporter into a polyamine importer by a single mutation. *Proceedings of the National Academy of Sciences* **109**, 16894-16899, doi:10.1073/pnas.1211831109 (2012).

6 Nasie, I., Steiner-Mordoch, S. & Schuldiner, S. New Substrates on the Block: Clinically Relevant Resistances for EmrE and Homologues. *Journal of Bacteriology* **194**, 6766-6770, doi:10.1128/JB.01318-12 (2012).

7 Lytvynenko, I., Brill, S., Oswald, C. & Pos, K. M. Molecular basis of polyspecificity of the Small Multidrug Resistance Efflux Pump AbeS from Acinetobacter baumannii. *Journal of Molecular Biology* **428**, 644-657, doi:10.1016/j.jmb.2015.12.006 (2016).

8 Paulsen, I. T., Brown, M. H., Dunstan, S. J. & Skurray, R. A. Molecular characterization of the staphylococcal multidrug resistance export protein QacC. *Journal of Bacteriology* **177**, 2827-2833, doi:10.1128/jb.177.10.2827-2833.1995 (1995).

9 Kazama, H., Hamashima, H., Sasatsu, M. & Arai, T. Characterization of the antiseptic-resistance gene qacEΔ1 isolated from clinical and environmental isolates of Vibrio parahaemolyticus and Vibrio cholerae non-O1. *FEMS Microbiol Lett* **174**, 379-384, doi:10.1111/j.1574-6968.1999.tb13593.x (1999).

10 Ploy, M.-C., Courvalin, P. & Lambert, T. Characterization of In40 of Enterobacter aerogenes BM2688, a Class 1 Integron with Two New Gene Cassettes, cmlA2 and qacF. *Antimicrobial Agents and Chemotherapy* **42**, 2557-2563, doi:10.1128/AAC.42.10.2557 (1998).

11 Heir, E., Sundheim, G. & Holck, A. L. The qacG gene on plasmid pST94 confers resistance to quaternary ammonium compounds in staphylococci isolated from the food industry. *Journal of Applied Microbiology* **86**, 378-388, doi:10.1046/j.1365-2672.1999.00672.x (1999).

12 Heir, E., Sundheim, G. & Holck, A. L. The Staphylococcus qacH gene product: a new member of the SMR family encoding multidrug resistance. *FEMS Microbiol Lett* **163**, 49-56, doi:10.1111/j.1574-6968.1998.tb13025.x (1998).

13 Bjorland, J., Steinum, T., Sunde, M., Waage, S. & Heir, E. Novel Plasmid-Borne Gene qacJ Mediates Resistance to Quaternary Ammonium Compounds in Equine Staphylococcus aureus, Staphylococcus simulans, and Staphylococcus intermedius. *Antimicrobial Agents and Chemotherapy* **47**, 3046-3052, doi:10.1128/AAC.47.10.3046-3052.2003 (2003).

14 Braga, T. M., Marujo, P. E., Pomba, C. & Lopes, M. F. S. Involvement, and dissemination, of the enterococcal small multidrug resistance transporter QacZ in resistance to quaternary ammonium compounds. *J Antimicrob Chemother* **66**, 283-286, doi:10.1093/jac/dkq460 (2011).
15 Parker, J. L. & Newstead, S. Structural basis of nucleotide sugar transport across the Golgi membrane. *Nature* **551**, 521-524, doi:10.1038/nature24464 (2017).
16 Nji, E., Gulati, A., Qureshi, A. A., Coincon, M. & Drew, D. Structural basis for the delivery of activated sialic acid into Golgi for sialyation. *Nat Struct Mol Biol* **26**, 415-423, doi:10.1038/s41594-019-0225-y (2019).
17 Tsuchiya, H. *et al.* Structural basis for amino acid export by DMT superfamily transporter YddG. *Nature* **534**, 417-420, doi:10.1038/nature17991 (2016).
