# Supplementary material for: The structural basis of promiscuity in small multidrug resistance transporters
Source: Nat Commun. 2020 Nov 27;11:6064. doi: 10.1038/s41467-020-19820-8 (PMC7695847; doi:10.1038/s41467-020-19820-8)
Supplement: Supplementary file 3 — Reporting Summary [file 41467_2020_19820_MOESM3_ESM.pdf]

nature research

Corresponding author(s): Randy B. Stockbridge

Last updated by author(s): Oct. 19, 2020

Reporting Summary

Nature Research wishes to improve the reproducibility of the work that we publish. This form provides structure for consistency and transparency in reporting. For further information on Nature Research policies, see our Editorial Policies and the Editorial Policy Checklist.

Statistics

For all statistical analyses, confirm that the following items are present in the figure legend, table legend, main text, or Methods section.

n/a	Confirmed	
☐	☒	The exact sample size (*n*) for each experimental group/condition, given as a discrete number and unit of measurement
☐	☒	A statement on whether measurements were taken from distinct samples or whether the same sample was measured repeatedly
☒	☐	The statistical test(s) used AND whether they are one- or two-sided<br>*Only common tests should be described solely by name; describe more complex techniques in the Methods section.*
☒	☐	A description of all covariates tested
☐	☒	A description of any assumptions or corrections, such as tests of normality and adjustment for multiple comparisons
☐	☒	A full description of the statistical parameters including central tendency (e.g. means) or other basic estimates (e.g. regression coefficient) AND variation (e.g. standard deviation) or associated estimates of uncertainty (e.g. confidence intervals)
☒	☐	For null hypothesis testing, the test statistic (e.g. *F*, *t*, *r*) with confidence intervals, effect sizes, degrees of freedom and *P* value noted<br>*Give P values as exact values whenever suitable.*
☒	☐	For Bayesian analysis, information on the choice of priors and Markov chain Monte Carlo settings
☒	☐	For hierarchical and complex designs, identification of the appropriate level for tests and full reporting of outcomes
☒	☐	Estimates of effect sizes (e.g. Cohen's *d*, Pearson's *r*), indicating how they were calculated

Our web collection on statistics for biologists contains articles on many of the points above.

Software and code

Policy information about availability of computer code

Data collection	surfe2r-n1 control v1.4.21 (Nanion Technologies). No custom code was used.
Data analysis	SHARP2.8, DIALS3.0, Refmac5.8.0257, Pymol2.3, Coot0.8.9.2, SHELX (version 2019/1), Phenix1.17.1, Molprobity4.5, Staraniso (webserver accessed October 2019-June 2020), Phaser-MR 2.7.0, Cytoscape3.8.1, Caver3.0, EFI-EST web tool (accessed April-May 2020). See methods for citations. No custom code was used.

For manuscripts utilizing custom algorithms or software that are central to the research but not yet described in published literature, software must be made available to editors and reviewers. We strongly encourage code deposition in a community repository (e.g. GitHub). See the Nature Research guidelines for submitting code & software for further information.

Data

Policy information about availability of data

All manuscripts must include a data availability statement. This statement should provide the following information, where applicable:

- Accession codes, unique identifiers, or web links for publicly available datasets
- A list of figures that have associated raw data
- A description of any restrictions on data availability

Atomic coordinates for the crystal structures have been deposited in the Protein Data Bank under accession numbers 6WK5, 6WK8, and 6WK9. Materials are available upon reasonable request. Source data are provided with this paper.

Field-specific reporting

Please select the one below that is the best fit for your research. If you are not sure, read the appropriate sections before making your selection.

☒ Life sciences ☐ Behavioural & social sciences ☐ Ecological, evolutionary & environmental sciences

For a reference copy of the document with all sections, see nature.com/documents/nr-reporting-summary-flat.pdf

Life sciences study design

All studies must disclose on these points even when the disclosure is negative.

Sample size	No statistical analysis other than standard error of measurement was used to show variance between replicates, so no sample size pre-determination was required. At least n=3 independent experiments were performed for each compound, which is standard for biochemical assays and sufficient for determining a mean and SEM. For SSM electrophysiology, data were collected from 3-4 independent sensor preparations, which were in turn prepared from 2-4 independent protein preparations. Because the electrode preparation is stable for a variable number of perfusions, the precise number of replicates varies for each compound. Experiments measuring radioactive uptake were independently repeated in triplicate.
Data exclusions	SSM electrophysiology: To compare measurements recorded on different sensors, currents were normalized relative to a reference compound, as described in the text. Currents elicited by the reference compound were measured both at the outset of the experiment and after collecting data on test compounds. If currents for the first and last perfusions of reference compound differed by more than 10%, this indicated that the amount of reconstituted protein had not remained stable over the course of the experiment, and data collected in this series were not used for further analysis. Exclusion criteria were pre-established.
Replication	Data were reproduced in multiple, independent experiments. SSM electrophysiology: Data were collected from 3-4 independent sensor preparations, which were in turn prepared from 2-4 independent protein preparations. Data collected by second independent experimenter for 3 reference compounds in agreement with data collected by main experimenter. Experiments measuring radioactive uptake were independently repeated in triplicate. All attempts at replication were successful.
Randomization	Samples were not allocated into experimental groups, so randomization is not applicable.
Blinding	Samples were not allocated into groups for data collection or analysis, so blinding is not applicable.

Reporting for specific materials, systems and methods

We require information from authors about some types of materials, experimental systems and methods used in many studies. Here, indicate whether each material, system or method listed is relevant to your study. If you are not sure if a list item applies to your research, read the appropriate section before selecting a response.

Materials & experimental systems

n/a	Involved in the study
☒	☐ Antibodies
☒	☐ Eukaryotic cell lines
☒	☐ Palaeontology and archaeology
☒	☐ Animals and other organisms
☒	☐ Human research participants
☒	☐ Clinical data
☒	☐ Dual use research of concern

Methods

n/a	Involved in the study
☒	☐ ChIP-seq
☒	☐ Flow cytometry
☒	☐ MRI-based neuroimaging
